# Low Power Long Duration Ablation for High Impedance Left Ventricular Summit PVCs

**DOI:** 10.1002/joa3.70185

**Published:** 2025-09-11

**Authors:** Yutsuki Tsukagoshi, Keijiro Nakamura, Shoma Kitano, Naohiko Sahara, Hidehiko Hara

**Affiliations:** ^1^ Division of Cardiovascular Medicine Toho University Ohashi Medical Center Tokyo Japan

**Keywords:** catheter ablation, high impedance, low power long duration, ventricular arrhythmia

## Abstract

Low‐power, long‐duration (LPLD) ablation (≤ 25 W for ≥ 60 s with 30 mL/min irrigation) was applied to high‐impedance (≥ 150 Ω) left ventricular summit PVC sites via the anterior interventricular vein. Power was escalated stepwise (10 W → 12 W → 15 W → 18 W) under strict safety criteria. Computer simulations demonstrated safe lesion formation at 20 W, whereas 30 W exceeded 100°C, predicting steam‐pop risk. This approach achieved 66.7% acute success without complications, highlighting LPLD as a promising option for challenging high‐impedance coronary venous ablation sites.

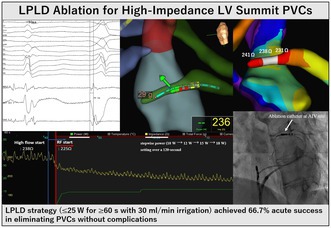

## 1 Introduction

Left ventricular summit premature ventricular contractions (PVCs) present unique challenges due to anatomical location and frequent coronary venous system access requirements. The coronary venous approach, particularly through the anterior interventricular vein (AIV), often encounters high impedance (HI) conditions exceeding 150 Ω, complicating radiofrequency ablation [1]. High impedance reduces current flow (Ohm's law), potentially compromising lesion formation while paradoxically increasing steam pop risk with higher power outputs [2]. This case series demonstrates successful low power long duration (LPLD) ablation for high impedance LV summit PVCs with computer simulation validation.

## 2 Case Presentation and Clinical Protocol

**FIGURE 1:**
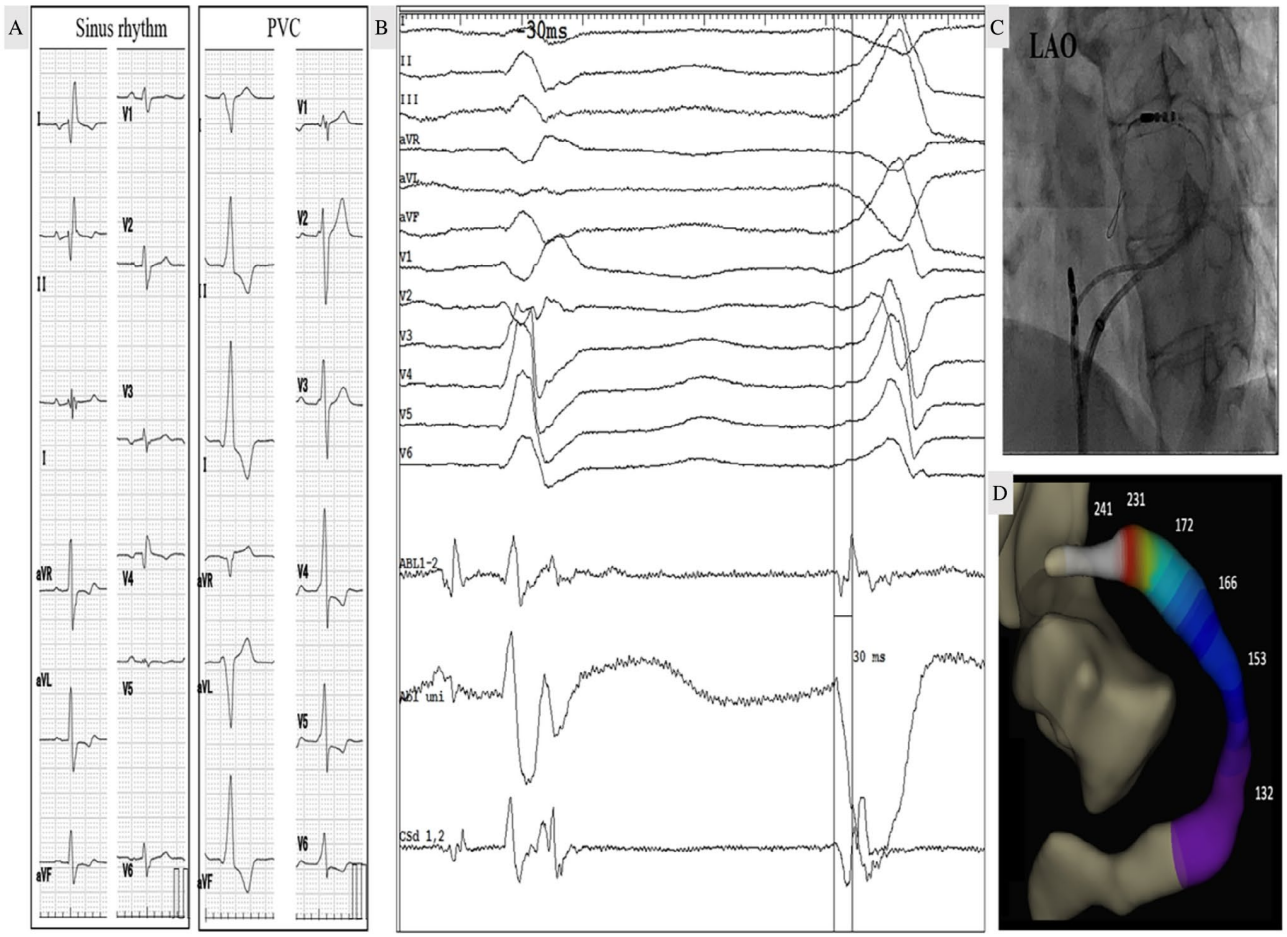
Electrocardiographic and procedural findings in a patient with high‐impedance left ventricular summit PVC. (A) Twelve‐lead electrocardiogram showing PVC morphology with inferior axis, left bundle branch block pattern, maximum deflection index (MDI) of 0.5 in V2, pseudo‐delta wave of 58 ms, and QaVL/QaVR ratio of 3.2, consistent with a left ventricular summit origin. (B) Intracardiac electrograms recorded from the anterior interventricular vein (AIV) showing 30 ms presystolic activation during PVC. (C) Fluoroscopic image (left anterior oblique view) showing the ablation catheter placed in the distal AIV (white arrow), approximately 15 mm from the GCV‐AIV junction. (D) Impedance map of the coronary venous system showing increased local impedance at distal AIV sites. A gradual rise in impedance was observed as the catheter advanced distally along the coronary vein.

A 68‐year‐old male with dilated cardiomyopathy presented with symptomatic PVCs. Holter monitoring revealed 25470 PVCs (20.5% burden) with LVEF 29%. Twelve‐lead ECG demonstrated inferior axis, left bundle branch block morphology PVCs suggesting LV summit origin, as shown in Figure 1A.

Electrophysiology study (EnSite X mapping system, TactiCath ablation catheter) revealed earliest activation 30 ms presystolic in the AIV, 15 mm distal from the GCV‐AIV junction (Figure 1B,C). Baseline impedance was 238 Ω. Figure 1D demonstrates the impedance map showing increased local impedance at distal AIV sites. Initial endocardial approaches failed to achieve PVC suppression.

### 2.1 LPLD Ablation Protocol

Given the high impedance environment, we implemented LPLD ablation defined as ≤ 25 W for ≥ 60 s with enhanced irrigation (≥ 30 mL/min). Radiofrequency energy was initiated at 10 W and gradually increased to 12, 15, and 18 W at approximately 30‐s intervals, provided that the following safety conditions were met: (1) no sudden impedance rise (> 10 Ω within 5 s), (2) no audible pops, (3) stable catheter position with adequate contact force, and (4) stable electrograms. This stepwise power escalation protocol allowed real‐time assessment of tissue response in high‐impedance coronary venous environments.

**FIGURE 2:**
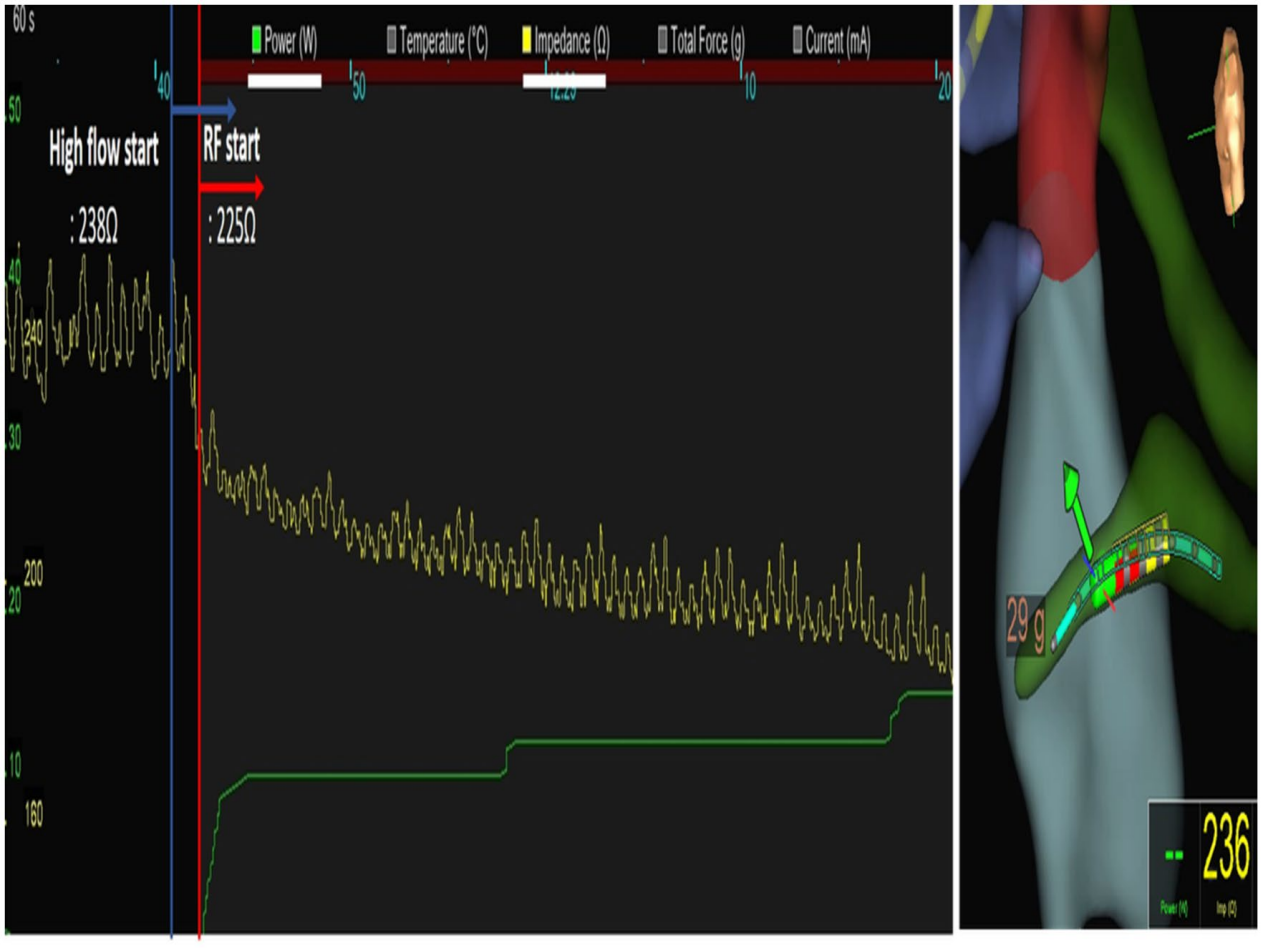
Ablation procedure and impedance monitoring. Impedance progression during LPLD ablation from 238 Ω baseline with gradual power escalation. PVC suppression occurred 8 s after ablation initiation at 211 Ω, followed by safe power escalation (10 W → 12 W → 15 W → 18 W) over 120 s without impedance rise.

PVC suppression occurred within 8 s at 211 Ω impedance. Power was safely escalated (10 W → 12 W → 15 W → 18 W) over 120 s, with final impedance of 179 Ω (total drop 59 Ω), as illustrated in Figure 2.

No procedural complications occurred. Three‐month follow‐up demonstrated sustained arrhythmia suppression.

## 3 Case Series Results

### 3.1 Additional Case Series

Six patients underwent LPLD ablation for high impedance coronary venous PVCs (Table 1). High impedance was pragmatically defined as baseline impedance ≥ 150 Ω, reflecting real‐world procedural challenges in coronary venous sites [1]. Baseline impedance ranged from 157 to 238 Ω. Demographics: mean age 63 ± 8 years, 3 males, 5 idiopathic PVCs, 1 ischemic cardiomyopathy. All cases involved LV summit/outflow tract origins requiring coronary venous access.

**TABLE 1:** Clinical characteristics and ablation outcomes of high‐impedance PVC sites (*n* = 6).

Case	Age	Sex	CM type	ECG morphology	HI site Info (location, impedance)	Ablation parameters at HI site (power, duration, CF, Imp drop)	Earliest lAT to QRS onset	Success at HI site	Final successful site	Complication
1	54	M	Idiopathic	Inferior, RBBB	AIV (188 Ω)	25 W, 151 s, 9 g, 64 Ω	42	Yes	AIV	None
2	65	F	Idiopathic	Inferior, RBBB	AIV (157 Ω)	16 W, 100 s, 24 g, 77 Ω	25	Yes	AIV	None
3	67	F	Idiopathic	Inferior, RBBB	AIV (162 Ω)	20 W, 60s, 15 g, 36 Ω	21	Yes	AIV	None
4	68	M	Idiopathic	Inferior, LBBB	AIV (238 Ω)	18 W, 120 s, 21 g, 59 Ω	48	Yes	AIV	None
5	53	F	Idiopathic	Inferior, RBBB	AIV (193 Ω)	15 W, 24 s, 19 g, 48 Ω	13	No	PA	None
6	72	M	ICM	Superior, RBBB	MCV (186 Ω)	20 W, 45 s, 19 g, 71 Ω	5	No	failure	None

Abbreviations: AIV, anterior interventricular vein; CF, contact force; CM, cardiomyopathy; HI, high impedance; ICM, ischemic cardiomyopathy; LAT, local activation time; MCV, middle cardiac vein; PA, pulmonary artery.

LPLD protocols (15–25 W, 24–151 s) achieved acute success at high impedance sites in 4/6 cases (66.7%). Two cases required alternative approaches: pulmonary artery success (Case 5) and unsuccessful ablation (Case 6). In these two cases, ablation parameters did not meet the predefined LPLD criteria due to clinical termination prompted by proximity to critical structures or early failure. These cases were classified as protocol deviations.

Mean impedance drop during ablation was 59 ± 15 Ω, reflecting improved electrode‐tissue coupling in high‐impedance environments. Despite prolonged ablation durations (up to 151 s), no steam pops, coronary complications, or dispersive pad burns occurred. At 3‐month follow‐up, 5/6 patients (83.3%) maintained PVC suppression.

## 4 Computer Simulation Validation

**FIGURE 3:**
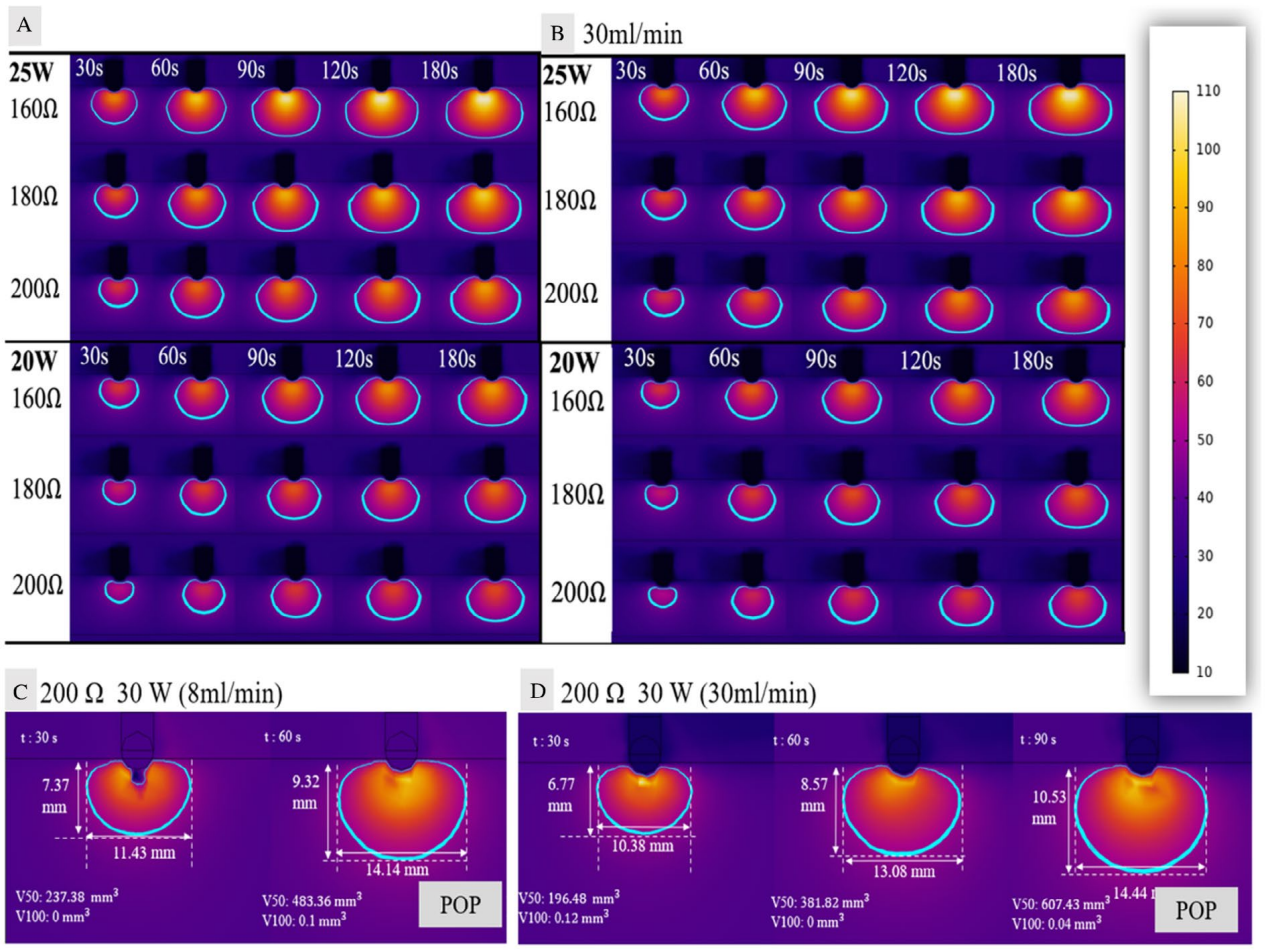
Computer simulation validation. (A, B) Lesion formation comparison at 200 Ω impedance: 20 W vs. 25 W with irrigation flow rates of 8 mL/min (A) and 30 mL/min (B). High impedance significantly affects lesion formation, but long duration application increases lesion size even at high impedance. (C, D) Temperature distribution at 30 W comparing irrigation flows. Thirty Watt exceeds critical temperature threshold after 60 s regardless of irrigation rate of 8 mL/min and after 90 s regardless of irrigation rate of 30 mL/min, demonstrating inherent steam pop risk at high power outputs in high impedance environments.

Computer simulation using established biophysical model [3, 4] with 10 g contact force validated the LPLD approach (detailed methodology in Supporting Information). The model employed COMSOL Multiphysics with Penne's bioheat transfer equation and temperature‐dependent electrical/thermal conductivities [4]. As demonstrated in Figure 3A,B, at 200 Ω impedance with 20 W/30 mL/min irrigation, adequate lesions formed without exceeding 100°C. However, 30 W protocols resulted in dangerous temperature elevation predicting steam pop formation, as shown in Figure 3C,D. Since actual AIV contact often exceeds 10 g, potentially increasing local impedance further, our conservative modeling supports the LPLD approach's safety margins. These findings confirm that power limitation (≤ 25 W) rather than enhanced irrigation alone ensures safety in high‐impedance environments.

## 5 Discussion

High impedance coronary venous sites traditionally pose significant ablation challenges. Our experience demonstrates LPLD modification provides an effective solution. The 238 Ω baseline impedance represents the upper range in distal coronary locations. According to Ohm's law (*p*=I^2^R), elevated impedance reduces current flow, potentially compromising lesion formation. However, our clinical and simulation data confirm that gradual energy delivery with enhanced cooling optimizes outcomes.

Our stepwise power escalation protocol is supported by experimental evidence. Qu et al. demonstrated baseline impedance ≥ 180 Ω significantly increases steam pop risk [2]. Although prior studies suggest impedance drops > 20 Ω may raise concerns, this threshold requires careful interpretation in high‐impedance environments where larger absolute drops reflect improved electrode‐tissue coupling [5, 6]. Our protocol focused on real‐time impedance trends, particularly abrupt drops or rises, rather than absolute thresholds.

Enhanced irrigation (30 mL/min) provides dual benefits: impedance reduction through improved electrode cooling and steam pop prevention [7]. The LPLD approach prioritizes duration over power intensity, using standard ablation equipment with reversible effects.

Contact force monitoring remains essential in anatomically constrained spaces like the AIV, helping avoid impedance elevation and vessel injury. Our systematic LPLD protocol demonstrates that safe ablation is achievable at sites > 150 Ω, potentially expanding treatment options for this challenging population, though larger studies are needed for validation. For example, the QDOT MICRO catheter, which delivers temperature‐controlled ablation at 4 or 15 mL/min, may not provide sufficient cooling in high‐impedance environments. Although the TactiFlex SE catheter provides contact force sensing and enhanced flexibility, contact monitoring remains essential in constrained venous spaces such as the AIV to avoid excessive force and impedance spikes.

## 6 Limitations

Small sample size limits generalizability. Three‐month follow‐up may be insufficient for long‐term assessment. The pragmatic LPLD definition (≤ 25 W, ≥ 60 s, ≥ 30 mL/min irrigation) aligns with prior reports but lacks universal standardization [8]. Our simulation assumed 10 g contact force; however, actual AIV forces often exceed this, potentially creating more challenging conditions than modeled. For cases refractory to LPLD, alternative approaches such as ethanol ablation may be considered, though this requires specialized expertise and was not evaluated in our study.

Coronary safety assessment relied on clinical monitoring without systematic angiography. Contact force measurements in coronary veins may be unreliable.

## 7 Conclusion

LPLD ablation represents a promising option for high‐impedance coronary venous PVC sites. This approach can be implemented with standard equipment, achieving 66.7% acute success without complications. Further studies with larger cohorts are warranted to validate long‐term efficacy and establish optimal protocol**s**.

## Consent

Written informed consent was obtained from the patient for publication of this case report and associated images.

## Conflicts of Interest

The authors declare no conflicts of interest.

## Supporting information


**Data S1:** joa370185‐sup‐0001‐Supinfo1@Supplemental Materials.docx.
