# Supplementary material for: Low Power Long Duration Ablation for High Impedance Left Ventricular Summit PVCs
Source: J Arrhythm. 2025 Sep 11;41(5):e70185. doi: 10.1002/joa3.70185 (PMC12423545; doi:10.1002/joa3.70185)
Supplement: Supplementary file 1 — Data S1: joa370185‐sup‐0001‐Supinfo1@Supplemental Materials.docx. [file JOA3-41-e70185-s001.docx]

**Supplemental Methods:**

**Computer Simulation Analysis Simulation Software and Model Construction:**

Computer simulations were performed using COMSOL Multiphysics 5.5.

A three-dimensional myocardial model was constructed with dimensions 90×80×40 mm, incorporating blood layers and perpendicular electrode placement (3.5 mm diameter, 10g contact force).

Governing Equations:

- Penne's bioheat transfer equation: ρcp∂T/∂t + ρcpu·∇T + ∇ - κ∇T = Q + Qbio

- Electrical field: ∇·(σ∇V) = 0

- Heat source: Q = σ|E|²

Model Parameters (37°C baseline):

- Myocardial electrical conductivity: 0.19 S/m (corresponding to 120Ω)

- Myocardial thermal conductivity: 0.53 W/(m·K) - Blood perfusion rate: 0.0028 s⁻¹

- Irrigation flow: 30 ml/min at 22°C

Temperature-Dependent Properties:

Electrical and thermal conductivities varied with temperature according to established functions [4], with impedance reaching minimum at ~100°C. Limitations: While contact force was fixed at 10g, clinical AIV ablation often involves higher forces due to anatomical constraints, potentially creating more challenging conditions than modeled.

Mesh Convergence Analysis: Mesh convergence was evaluated using temperature and lesion size as assessment criteria. Four mesh sizes (coarse, medium, regular, refined) were tested under 15W for 120s conditions. The regular mesh (154,913 domain elements, 14,674 boundary elements) achieved <1% error in both temperature and lesion size compared to refined mesh, confirming adequate convergence while maintaining computational efficiency [4].

Boundary Conditions:

- Upper electrode: Active electrode with applied potential V = U0 - Lower electrode: Ground (V = 0V) - Remaining boundaries: Electrically insulated (I = 0A) - Initial myocardial/blood temperature: 37°C - Electrode temperature: 22°C - Blood flow: 0.1 m/s from left to right boundary

- Irrigation flow: 13 ml/min (equivalent to 30 ml/min clinical setting)

- Model cross-section: Thermally insulated for lesion observation

Model Validation:

Simulation accuracy was validated against in vitro experimental data. For 50W bipolar ablation in 17.6mm myocardium, simulation predicted lesion areas of 7.7×7.2mm² (endocardium) and 7.6×7.0mm² (epicardium), compared to experimental values of 6.3×5.8mm² and 5.9×5.3mm² respectively. The slight overestimation (<20%) is acceptable considering idealized simulation conditions versus experimental uncertainties[4]. Note: While our study focused on unipolar ablation, the validated model framework and temperature-impedance relationships remain applicable.

4. Sun Y, Zhu X, Chen W, Weng W, Nakamura K. Computer simulation of low-power and long-duration bipolar radiofrequency ablation under various baseline impedances. Med Eng Phys. 2024;131:104226.
